# Bioinformatics and experimental studies of atherosclerosis-related endothelial dysfunction genes in renal calculi and exploration of their molecular mechanisms

**DOI:** 10.1371/journal.pone.0354595

**Published:** 2026-08-04

**Authors:** Aina Li, Chenjing Liu, Xiangshen Liu, Lei Chen, Dong Wang, Changyan Zhu

**Affiliations:** 1 Department of Cardiology, the First Affiliated Hospital of Fujian Medical University, FuZhou, China; 2 900th Hospital of PLA Joint Logistic Support Force, FuZhou, China; Public Library of Science, UNITED KINGDOM OF GREAT BRITAIN AND NORTHERN IRELAND

## Abstract

Patients with kidney stones (KS) often have an increased risk of atherosclerosis (AS). Because endothelial dysfunction (ED) is closely associated with AS, its role in KS remains unclear. This study aimed to examine the roles and mechanisms of AS-related ED genes in KS. Three datasets (GSE73680, GSE117518, and GSE132651) were analyzed. Differential expression analysis was conducted to identify differentially expressed genes (DEGs). To identify potential biomarkers, least absolute shrinkage and selection operator (LASSO) regression analysis and expression validation were conducted. Further analyses including GeneMANIA, gene set enrichment analysis (GSEA), examination of biomarkers within immune cells and subcellular localization analysis, molecular regulatory network analysis, tissue specificity analysis, and competing endogenous (ceRNA) network analysis were employed to comprehensively explore the functions and regulatory mechanisms of the identified biomarkers. Moreover, drug prediction analysis was conducted. Finally, reverse transcription quantitative polymerase chain reaction (RT-qPCR) was proceeded to verify the expression levels of the biomarkers. A total of 22 DEGs associated with KS and AS were identified. Lasso regression selected 4 candidate biomarkers (MMP10, UCHL1, NEK2, and HEY1), among which UCHL1 and NEK2 were validated as key biomarkers. GeneMANIA and GSEA analyses uncovered the potential involvement of these biomarkers in cell adhesion molecules, focal adhesion, and lysosome pathways. Analysis of immune cells and subcellular localization provided insight into the biological functions and intracellular distribution of the biomarkers. Transcription factor regulatory network and ceRNA network analyses elucidated potential upstream regulatory mechanisms. Drug prediction analysis identified 17 potential drugs, including pazopanib and palbociclib, that may target NEK2. RT-qPCR demonstrated that *NEK2* was significantly overexpressed in KS samples. This study identified biomarkers associated with KS and AS and comprehensively analyzed their molecular regulatory networks. These findings provide novel understandings of the molecular mechanism underlying KS and lay the foundation for future personalized treatment and drug development.

## Introduction

Kidney stones (KS) are hard crystalline deposits formed by the aggregation of minerals and salts (e.g., calcium oxalate, calcium phosphate, and uric acid) in the kidneys [1]. As a global public health problem, the prevalence of KS is rising, particularly in industrialized nations, where dietary factors are recognized as major contributors to the rising incidence of urolithiasis [2]. In China, the prevalence of KS among adult is approximately 7.54% [3]. Common stone components include calcium oxalate, calcium phosphate, and uric acid. The pathogenic mechanisms underlying KS include urinary supersaturation, a deficiency of crystallization inhibitors, and urinary tract infections [4]. Clinical manifestations may include flank pain, hematuria, and urinary frequency. Treatment strategies include medical clearance, extracorporeal shock wave lithotripsy, and surgical interventions [5]. The clinical management of KS faces challenges such as complex stone morphology, technical limitations of therapeutic approaches, postoperative complications, and high recurrence rates [6]. Addressing these challenges requires technological innovation, personalized treatment strategies, and enhanced patient education [7].

Emerging evidence suggests a potential association between KS and atherosclerosis (AS), mediated by shared pathophysiological mechanisms, including dysregulated calcium metabolism, chronic inflammation, and oxidative stress [8]. Epidemiological studies have demonstrated that patients with KS have a significantly elevated risk of AS compared with individuals without KS. For instance, a cohort study of 1,027 patients with KS revealed a twofold increase in the prevalence of abdominal aortic calcification (AAC) compared to controls [9]. Elucidating the precise relationship between KS and AS is critical for understanding the pathogenesis of KS and may provide novel therapeutic avenues to improve clinical outcomes and reduce recurrence [10].

Endothelial dysfunction (ED), characterized by impaired vasodilation, angiogenesis, and barrier function, is an early pathogenic hallmark of cardiovascular disease. ED is strongly associated with metabolic disorders such as diabetes, obesity, and metabolic syndrome conditions that are also linked to cardiovascular morbidity [11]. The pivotal roles of oxidative stress and inflammation in ED pathogenesis are well established, and ED is recognized as an early stage of vascular disease and atherogenesis, predisposing individuals to clinical complications during the preclinical phase [12]. In AS, ED contributes to endothelial injury, lipid deposition, inflammatory plaque formation, smooth muscle cell proliferation, fibrous cap development, and plaque instability leading to thrombosis [13]. Epidemiological data indicate that KS patients have an increased risk of ED and cardiovascular disease, suggesting a shared pathological basis [14]. KS-associated local inflammation releases cytokines (e.g., IL-6, TNF-α) that activate endothelial inflammatory pathways (e.g., NF-κB), upregulate adhesion molecules (e.g., VCAM-1 and ICAM-1), and promote leukocyte infiltration. ED further exacerbates renal inflammation and promotes stone formation [15]. Despite the established interplay between ED and KS, the molecular mechanisms by which AS-related ED genes in KS development remain incompletely understood. Deciphering these mechanisms is essential for the development of novel preventive and therapeutic strategies to improve KS prognosis.

In this study, we performed differential gene expression analysis between KS and normal tissues and applied least absolute shrinkage and selection operator (LASSO) regression-based machine learning to identify and validate key AS-related ED biomarkers. We further delineated upstream regulatory networks and predicted potential therapeutic agents, thereby providing novel insights into the molecular mechanisms, biomarker identification, and personalized treatment of KS.

## Materials and methods

### Data collection

The datasets, GSE73680-GPL17077, GSE117518-GPL21827, and GSE132651-GPL96, were obtained from the Gene Expression Omnibus (GEO) database (https://www.ncbi.nlm.nih.gov/). The training cohort (GSE73680) comprised 29 renal papillary (RP) tissue samples from patients with KS and 6 RP tissue samples from controls [16], whereas the validation cohort (GSE117518) included 3 RP tissue samples from patients with KS and 3 RP tissue samples from controls. Both datasets consisted of RP tissues from patients and normal controls, which closely matched the specific lesion site investigated in this study. The GSE132651 dataset comprised blood endothelial cell samples from 6 patients with AS exhibiting normal endothelial function and 13 patients with AS and ED, focusing on genes associated with AS-related ED [17].

### Difference expression analysis

In GSE73680, differential expression analysis between KS and control samples was performed using the limma package (v3.54.0) to identify differentially expressed genes (DEGs) (P < 0.05 and |log_2_FC| > 1) [18].Subsequently, the limma package (v3.54.0) was also used to obtain DEGs in GSE132651 between AS and control groups (P < 0.05 and |log_2_FC| > 0.5). Volcano plots and heat maps were generated using the ggplot2 (v3.4.1) and ComplexHeatmap packages (v2.15.1), respectively, to visualize the results [19,20].

### Identification and functional annotation of key genes

To identify genes associated with AS-related ED in KS, the intersection of DEGs was determined using the ggVenn package (v1.1.0), and the overlapping genes were defined as key genes [21]. Gene Ontology (GO) and Kyoto Encyclopedia of Genes and Genomes (KEGG) enrichment analyses were then performed, with an adjusted P value < 0.05 considered statistically significant.

### Identification of biomarkers

To identify potential biomarkers, the glmnet package (v4.1.8) was utilized to perform LASSO regression analysis on the key genes, and the selected genes were defined as candidate biomarkers [22]. Subsequently, in GSE73680 and GSE117518, the expression levels of the candidate biomarkers in KS and control samples were compared using the Wilcoxon test. Candidate biomarkers that exhibited consistent and statistically significant expression pattern in both datasets were defined as validated biomarkers.

### Functional enrichment of biomarkers

To identify genes functionally associated with the biomarkers, GeneMANIA (http://www.genemania.org/) was used to predict interacting genes and their related biological functions. Following this, expression levels of the biomarkers in GSE73680 were then dichotomized into high- and low-expression groups based on the median value. Subsequently, differential expression analysis was performed between the high- and low-expression groups in the training cohort to calculate logFC values for all genes. The genes were ranked in descending order according to their logFC values to generate a gene-ranked list for each biomarker. The GSVA package (v1.32.0) was used to conduct gene set enrichment analysis (GSEA) for the biomarkers, based on the c2.cp.v2023.1.Hs.symbols.gmt gene set from the Molecular Signatures Database (MSigDB) (http://www.gsea-msigdb.org/) [23].

### Analysis of biomarkers in immune cells and their subcellular localization

The expression of the biomarkers in immune cells was analyzed using the Human Protein Atlas (HPA) database (https://www.proteinatlas.org/) to determine their expression patterns across immune cell types. Additionally, the subcellular localization of the biomarkers was analyzed using the Universal Protein Resource (UniProt) (https://www.uniprot.org/) and the HPA database.

### Molecular regulatory networks and tissue-specific analysis

To identify the transcription factors (TFs) that regulate the biomarkers in KS, the NetworkAnalyst platform (https://www.networkanalyst.ca/) was used to predict TF-biomarkers interaction. The TF-biomarker regulatory network was constructed using the Cytoscape software (v3.8.2) [24]. To further characterize the tissue-specific expression of the biomarkers, the Genotype-Tissue Expression (GTEx) portal (https://gtexportal.org/home/) was used to analyze their expression profiles across various tissues.

### Competing endogenous RNA (ceRNA) network and drug prediction

To investigate the molecular regulatory mechanisms of the biomarkers, the Encyclopedia of RNA Interactomes (ENCORI) (http://starbase.sysu.edu.cn/), miRcode (https://www.broadinstitute.org/ccle/miRcode), and miRwalk (http://mirwalk.umm.uni-heidelberg.de/) databases were used to predict upstream miRNAs targeting these biomarkers. The predicted results from the three databases were intersected to identify shared miRNAs. Furthermore, ENCORI was used to predict long noncoding RNAs (lncRNAs) that regulate the identified miRNAs, with inclusion limited to interactions supported by at least five experimental validations. Cytoscape software (v3.8.2) was then applied to construct the lncRNA-miRNA-biomarker regulatory network. The limma package (v3.54.0) was applied to perform differential expression analysis of the lncRNA expression matrix in GSE117518 between the KS and control groups, with P < 0.05 and |log_2_FC| > 0.5 set as the screening criteria. The intersection between the predicted lncRNAs and the differentially expressed lncRNAs was analyzed to validate the reliability of the lncRNA predictions.

To explore potential therapeutic agents for KS, candidate drugs targeting the biomarkers were identified using the Drug-Gene Interaction Database (DGIdb) (http://www.dgidb.org/) and the Comparative Toxicogenomics Database (CTD) (http://ctdbase.org), both available through the NetworkAnalyst platform (https://www.networkanalyst.ca/). The gene-drug network was visualized using the Cytoscape software (v3.8.2).

### Reverse transcription quantitative polymerase chain reaction (RT-qPCR)

The expression levels of the biomarkers were validated by RT-qPCR. A total of 5 pairs of KS and control tissue samples collected between January 2023 and April 2025, were obtained from the 900th Hospital of PLA Joint Logistic Support Force. This study was approved by the Ethics Committee of the 900th Hospital of PLA Joint Logistic Support Force and was conducted in accordance with the Declaration of Helsinki (approval number: 2025–092). Written informed consent was obtained from all participants. Patient data were accessed on August 11, 2025. No authors had access to information that could identify individual participants during or after data collection. The samples were processed for RNA isolation on August 23, 2025. Total RNA was extracted from the ten samples using TRIzol reagent (Ambion, USA) according to the manufacturer'ss instructions. RNA concentrations were measured using a NanoPhotometer N50. Complementary DNA (cDNA) was synthesized using the SureScript First Strand cDNA Synthesis Kit (Servicebio, China). Quantitative PCR was performed using a CFX Connect Thermal Cycler (Bio-Rad, USA). GAPDH was used as the internal reference gene for RT-qPCR. Relative mRNA expression levels were calculated using the 2^-ΔΔCT^ method. The primer sequences are provided in S1 Table.

### Statistical analysis

All data were analyzed using the R software. The Wilcoxon test was used to compare differences between groups, with P < 0.05 considered statistically significant.

## Results

### A total of 22 key genes were obtained

**Fig 1:**
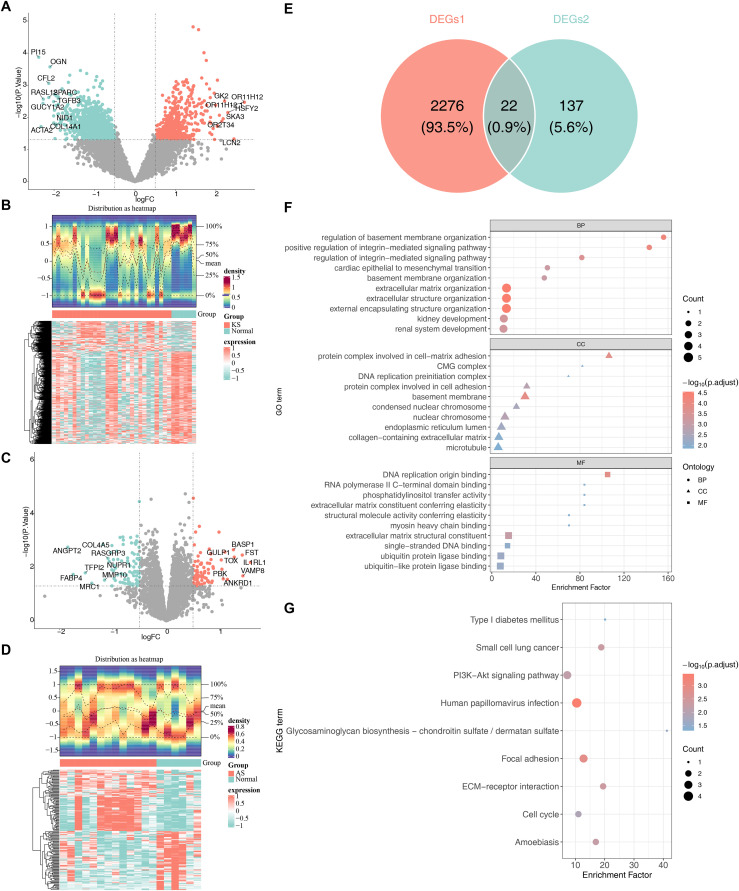
Differentially expressed genes between the KS group and the control group. (A) Volcano plot of differential expression analysis for GSE73680. (B) Heatmap of differential expression analysis for GSE73680. (C) Volcano plot of differential expression analysis for GSE132651. (D) Heatmap of differential expression analysis for GSE132651. (E) Venn diagram showing the intersection between DEGs1 and DEGs2. (F) GO enrichment analysis. (G) KEGG enrichment analysis.

A total of 2,298 DEGs (S2 Table) was identified between KS and control samples, of which 569 were upregulated and 1,729 were downregulated (Fig 1A and 1B). A total of 159 DEGs (S3 Table) were identified between AS and control groups, including 81 upregulated and 78 downregulated genes (Fig 1C and 1D). Moreover, 22 key genes were obtained by intersecting the two DEG lists (Fig 1E). Functional enrichment analysis revealed that these key genes were primarily associated with GO terms related to renal system development, kidney growth, the external wrapping tissue, extracellular matrix organization, basement membrane components, microtubule structure, collagen-containing ECM, endoplasmic reticulum lumen, condensed chromatin, chromosomes with intense staining, basement membrane, extracellular matrix structural components and myosin heavy chain binding etc. (Fig 1F). In addition, KEGG pathway analysis indicated enrichment in pathways such as amebiasis, cell cycle, lesion adhesion, glycosaminoglycan biosynthesis-chondroitin sulfate/dermatan sulfate, and type 1 diabetes mellitus (Fig 1G).

### UCHL1 and NEK2 were identified as biomarkers

**Fig 2:**
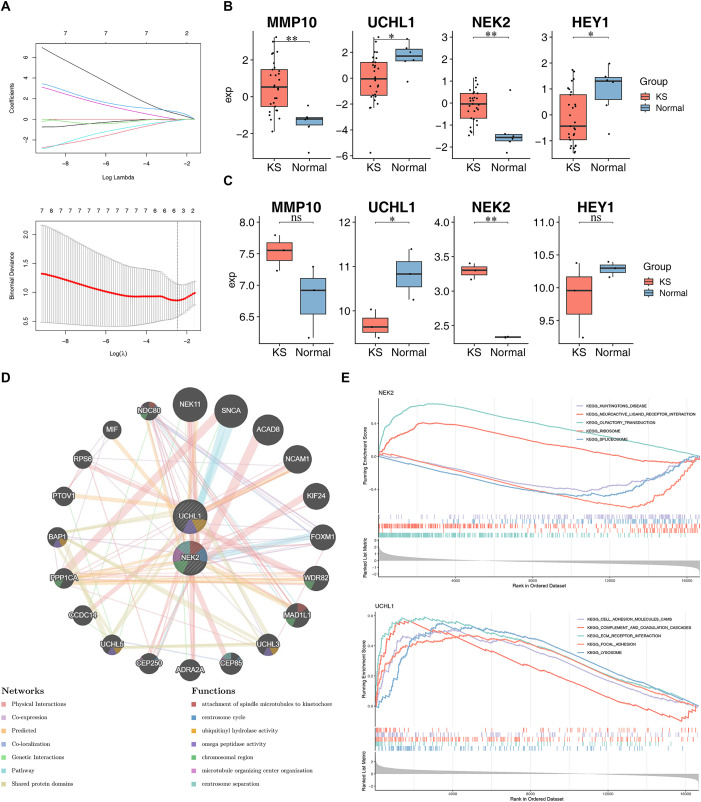
UCHL1 and NEK2 identified as diagnostic genes. (A) LASSO regression analysis. (B) Expression levels of candidate biomarkers in GSE73680. (C) Expression levels of candidate biomarkers in GSE117518. (D) GeneMANIA analysis of biomarkers. (E) GSEA enrichment analysis of biomarkers.

LASSO regression analysis of the 22 key genes yielded 4 candidate biomarkers, namely MMP10, UCHL1, NEK2, and HEY1 (Fig 2A). Concurrently, among these 4 candidate biomarkers, UCHL1 and NEK2 exhibited consistent and statistically significant differential expression in both datasets. Therefore, UCHL1 and NEK2 were selected as the final biomarkers (Fig 2B and 2C).

### High functional similarity between the biomarkers

To identify additional genes associated with UCHL1 and NEK2, GeneMANIA was used to construct an interactive network diagram comprising 20 predicted genes. Within this network diagram, most interactions were attributed to physical interactions, accounting for 77.64% of the total interactions. Co-expression interactions represented 8.01%, predicted interactions accounted for 5.37%, co-localization interactions comprised 3.63%, genetic interactions accounted for 2.87%, pathway-related interactions represented 1.88%, and shared protein domains accounted for a mere 0.6%. The associated functional pathways encompassed a variety of cellular processes such as interaction between spindle microtubules and kinetochores, regulation of the centrosome cycle, ubiquitin hydrolases activity, omega peptidases activity, chromosome region organization, and separation (Fig 2D).

Furthermore, GSEA enrichment analysis demonstrated that NEK2 was primarily enriched in positively correlated pathways, including Huntington's disease, neuroactive ligand-receptor interactions, and negatively correlated pathways including ribosome and spliceosome pathways (S4 Table). UCHL1 was positively associated with pathways involving cell adhesion molecules, complement and coagulation cascades, ECM receptors interactions, focal adhesion, and the lysosomal pathway (Fig 2E, S5 Table).

### Strong correlation between immune cells and biomarkers

**Fig 3:**
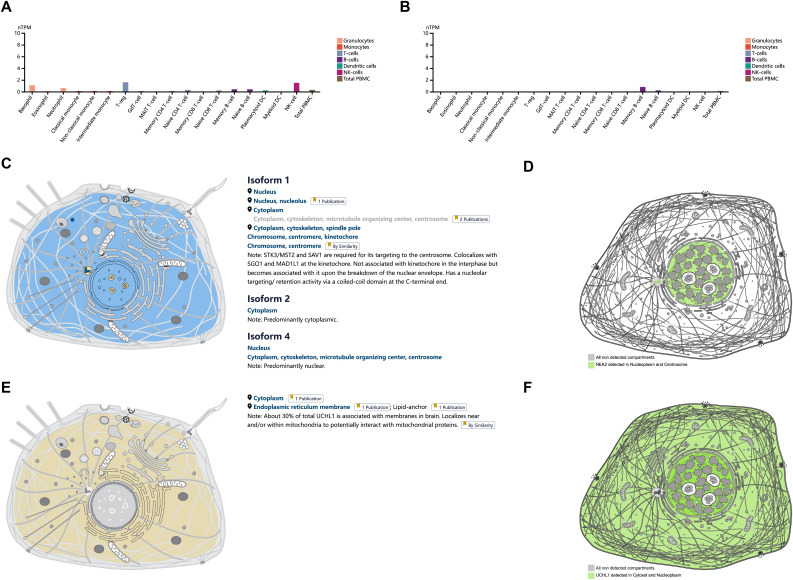
Analysis of NEK2 and UCHL1 expression in immune cells and their subcellular localization. (A) Expression level of NEK2 in immune cells. (B) Expression level of UCHL1 in immune cells. The expression levels of NEK2 and UCHL1 in immune cells were standardized based on data from the HPA database. NEK2 exhibited moderate-to-high expression across multiple immune cell types. (C, D) Subcellular localization of NEK2. (E, F) Subcellular localization of UCHL1.

Biomarker expression across immune cell types were analyzed. The results showed that NEK2 exhibited moderate-to-high expression levels across multiple immune cell lineages and was highly expressed in regulatory T cells, natural killer cells, and basophils (Fig 3A). UCHL1 expression, on the other hand, was detected only in memory B-cells and naive B-cells (Fig 3B).

Furthermore, analysis of the subcellular localization of these biomarkers demonstrated that NEK2 protein was predominantly localized to the nuclear matrix and centrosomes, which is consistent with its established role in cell cycle regulation (Fig 3C and 3D). Conversely, UCHL1 protein was primarily localized to the nuclear matrix and cytoplasm (Fig 3E and 3F).

### Potential regulatory mechanisms of biomarkers

**Fig 4:**
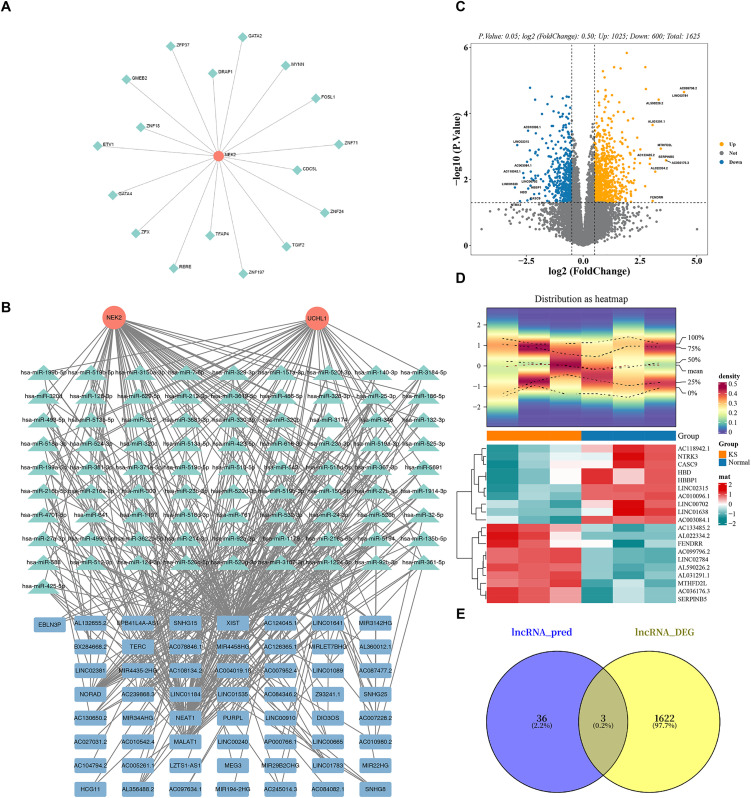
Molecular regulatory networks. (A) TF-biomarker network. (B) lncRNA-miRNA-biomarker network. (C) Volcano plot of differentially expressed lncRNAs between the KS and control groups. (D) Heatmap of differentially expressed lncRNAs between the KS and control groups. (E) Venn diagram of intersecting lncRNAs.

To further elucidate the regulatory roles of the biomarkers, TF prediction analysis was performed. The results identified 17 TFs predicted to regulate NEK2 expression, potentially influencing the formation and progression of AS (Fig 4A). In addition, 66 miRNAs were identified as potential regulators of NEK2, which were further regulated by 39 upstream lncRNAs. For UCHL1, 37 miRNAs were predicted to regulation its expression, and these miRNAs were controlled by 31 upstream lncRNAs. The lncRNA-miRNA-biomarker regulatory network is shown in Fig 4B and highlights several regulatory axes, including EBLN3P-hsa-miR-186-5p-NEK2, XIST-hsa-miR-199b-5p-UCHL1, AC010980.2-hsa-miR-361-5p-NEK2, and LINC01089-hsa-miR-3184-5p-UCHL1. Differential expression analysis of the lncRNA expression matrix from GSE117518 was conducted between the KS and control groups, yielding 1,625 differentially expressed lncRNAs, including 1,025 upregulated and 600 downregulated lncRNAs (Fig 4C and 4D; S6 Table). Among the 39 predicted lncRNAs involved in the NEK2 regulatory network, intersection analysis with the 1,625 differentially expressed lncRNAs identified three overlapping lncRNAs: AC108134.2, SNHG15, and MIR4458HG (Fig 4E).

### Tissue-specific analysis and drug prediction

**Fig 5:**
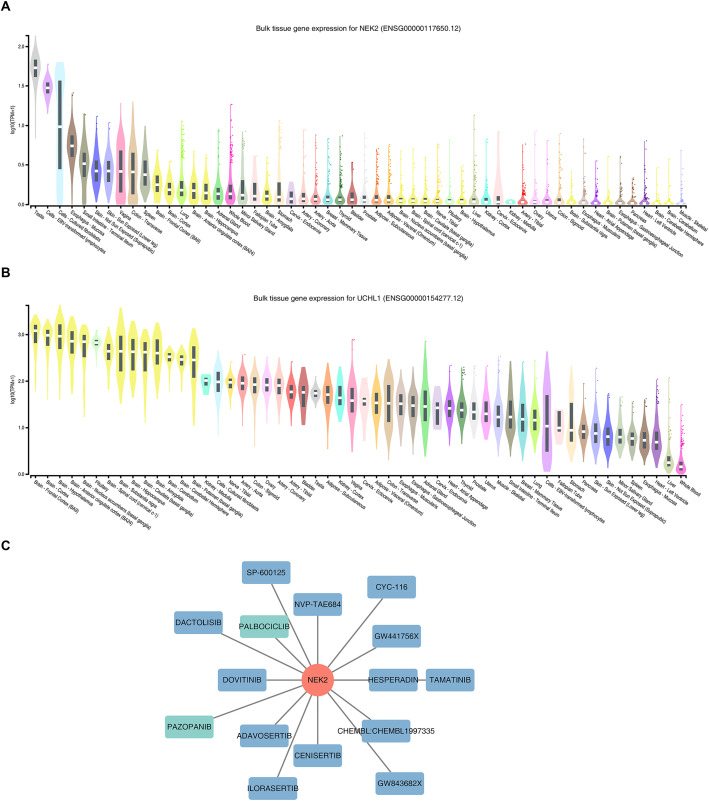
Tissue specific expression and drug prediction analysis. (A) Tissue-specific expression of NEK2. (B) Tissue-specific expression of UCHL1. (C) Drug prediction for the biomarkers.

Analysis of the GTEx database was performed to further characterize the tissue-specific expression of the biomarkers. NEK2 exhibited higher expression levels in the testes, lymphocytes, and fibroblasts (Fig 5A). Similarly, UCHL1 showed higher expression in brain tissue, kidney tissue, and fibroblasts (Fig 5B). Subsequently, a total of 15 drugs were predicted to target and regulate NEK2, among which 2 drugs had been approved (pazopanib and palbociclib) (Fig 5C).

### NEK2 was over-expressed in KS samples

**Fig 6:**
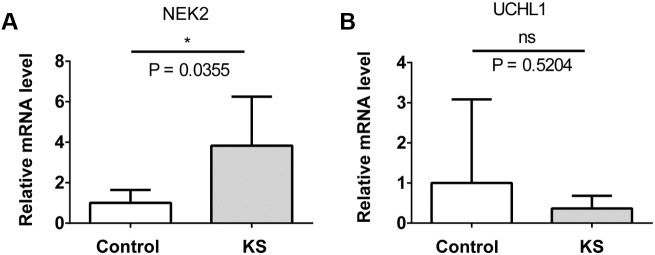
Validation of NEK2 and UCHL1 expression levels. (A) Differential expression of NEK2. (B) Differential expression of UCHL1.

RT-qPCR analysis demonstrated that NEK2 was significantly upregulated in KS samples (P = 0.0355) (Fig 6A). In contrast, UCHL1 expression showed no statistically significant difference between the two groups (P > 0.05), although a trend toward lower expression was observed in KS samples (Fig 6B).

## Discussion

Globally, both KS and AS have high incidence rates and exhibit substantial population heterogeneity. Pronounced differences in genetic backgrounds, living environments, and metabolic traits exist among populations, including those in Western countries and Asia [25]. International transcriptomic and genome-wide association studies have consistently identified dysregulated calcium metabolism, inflammation, oxidative stress, and aberrant cell adhesion as core pathogenic pathways in kidney stone disease (KSD). The strong concordance between these global findings and our bioinformatic results reflects the cross-population conservation of these molecular mechanisms. Currently, KS is recognized as a systemic disorder and is closely associated with cardiovascular diseases, including AS [26,27]. The generation of reactive oxygen species and the subsequent development of oxidative stress are common pathological features shared by both AS and KS. However, the precise relationship between AS and KS remains to be fully elucidated. In the present study, we identified NEK2 and UCHL1 as shared biomarkers of KS and AS.

NEK2, a serine/threonine kinase that regulates centrosome duplication and mitotic progression, was significantly overexpressed in KS tissues. This finding is consistent with studies demonstrating that oxidative stress and calcium oxalate crystals induce centrosome amplification in renal tubular cells, thereby promoting aberrant cell proliferation and crystal adhesion [28]. The enrichment of NEK in pathways such as Huntington'ss disease further suggests shared mechanisms involving oxidative damage and mitochondrial dysfunction in KSD. Conversely, UCHL1, a deubiquitinating enzyme involved in proteostasis and apoptosis, may mitigate renal injury by stabilizing anti-apoptotic proteins under conditions of metabolic stress. Although UCHL1 has not previously been linked to KSD, its upregulation in diabetic nephropathy suggests a conserved role in cellular stress adaptation, potentially counteracting tubular cell death triggered by crystal-induced inflammation [29]. In this study, based on the epidemiological association between KSD and ED, bioinformatic analysis of GEO datasets identified NEK2 and UCHL1 as key biomarkers, which were validated by RT-qPCR. Functional enrichment analysis indicated that both genes are involved in processes critical to renal tubular injury and fibrosis. Regulatory network analysis further identified their upstream transcriptional regulators and potential drug candidates. These findings suggest that NEK2 and UCHL1 may serve as dual biomarkers of disease progression and that targeting NEK2 or modulating UCHL1 activity may represent potential therapeutic strategies. This study provides a foundation for precision therapies targeting ED-associated pathways in KSD, with the potential to reduce stone recurrence and cardiovascular risk.

UCHL1, a deubiquitinating enzyme primarily involved in protein degradation and cellular signaling, has been extensively studied in neurological disorders and cancer. Recent studies have demonstrated a crucial role of UCHL1 in maintaining endothelial homeostasis. It regulates lung endothelial cell permeability [30], promotes the angiogenesis of brain microvascular endothelial cells by stabilizing SOX17 [31], and contributes to pulmonary hypertension through the deubiquitination of AKT [32]. In the kidney, UCHL1 deficiency can lead to proteinuria, urinary retention, and hypotension, suggesting an indispensable role in renal homeostasis [33]. NEK2, a member of the NIMA-related serine/threonine kinase family located on chromosome 1q32.2-1q41, contains an N-terminal kinase domain and a C-terminal regulatory domain. NEK2 localizes to both the cytoplasm and nucleus and plays a critical role in mitosis [34]. Several studies have reported the involvement of NEK2 in lipopolysaccharide-induced endothelial barrier dysfunction [35,36]. Inhibition of NEK2 reduces endothelial permeability, reactive oxygen species generation, and phosphorylation of key inflammatory proteins in lipopolysaccharide-treated cells [36]. In clear cell renal cell carcinoma, NEK2 overexpression is associated with worse clinical outcomes, higher mutation burden, and increased infiltration of various immune cells. Nevertheless, the involvement and functions of NEK2 and UCHL1 in AS and KS remain unclear.

Although no direct studies have investigated UCHL1 or NEK2 in the pathogenesis of KSD, their established roles in oxidative stress, inflammation, and renal tubular repair suggest potential involvement. UCHL1 may modulate oxidative stress and inflammation during stone formation, whereas NEK2 may influence cell cycle regulation in tubular epithelial cells or fibrosis-related pathways [37]. Given that aberrant proliferation and impaired repair of renal tubular epithelial cells are pivotal processes in KS formation, and that ED and abnormal proliferation contribute to AS progression, our findings suggest that NEK2 may act as a molecular link between these two diseases by modulating cell cycle dynamics. Of the 17 predicted therapeutic agents, most have no direct clinical application in KSD. However, mechanistic plausibility supports potential drug repurposing. CYC-116, an Aurora A/B and VEGFR2 inhibitor used in cancer therapy, could theoretically target tubular cell proliferation if such proliferation contributes to stone formation [38]. SP-600125, a JNK/c-Jun inhibitor, may attenuate stone-related injury by suppressing apoptosis, inflammation, and oxidative stress [39]. Pazopanib, a multitarget tyrosine kinase inhibitor (VEGFR/PDGFR), may inhibit angiogenesis or inflammatory pathways if these mechanisms are implicated in KSD. RT-qPCR analysis revealed upregulated NEK2 expression in stone formers, identifying this ED-related gene as a potential novel biomarker. TF regulatory network analysis identified FOSL1, a TF involved in cell proliferation and inflammatory responses, as a potential upstream regulator of NEK2 [40]. These findings suggest that NEK2 dysregulation may be driven by conserved pro-inflammatory and proliferative transcriptional programs. ceRNA network analysis further indicated that NEK2 is regulated by specific lncRNAs and miRNAs. Notably, NEAT1 has been implicated in renal inflammation, fibrosis, and vascular lesions [41,42]. Tissue expression analysis showed high NEK2 expression in fibroblasts, suggesting its involvement in fibroblast proliferation, activation, and subsequent fibrotic processes. Excessive fibroblast activation and fibrosis are hallmark features of maladaptive repair in KS and progression of atherosclerotic plaques in AS. Further mechanistic studies and clinical validation are essential to translate these findings into therapeutic strategies.

This study identified UCHL1 and NEK2 as novel biomarkers for KS through integrated bioinformatics analysis and experimental validation. Comprehensive analyses revealed their roles in regulating centrosome dynamics (NEK2) and proteostasis (UCHL1), processes that are critical to renal tubular injury and calcium crystal adhesion. The PI3K-Akt pathway and ECM remodeling emerged as key mechanisms linking ED to KS progression. Furthermore, drug prediction analysis, including the identification of pazopanib as a potential NEK2-targeting agent, provides a theoretical foundation for repurposing existing therapies to mitigate stone recurrence and fibrosis [43]. These findings contribute to a deeper understanding of the molecular mechanisms underlying related diseases, as well as to biomarker identification and therapeutic development.

This study has several limitations that warrant consideration. First, UCHL1 did not reach statistical significance in our cohort, which may be attributable to the small sample size and tissue heterogeneity. Second*,* NEK2 and UCHL1 were preliminary biomarkers identified through bioinformatics analysis, and their expression was evaluated only at the transcriptional level in a relatively small cohort. Western blotting and immunohistochemistry should be performed to assess their protein expression levels in an independent clinical cohort with a larger sample size. Third, although their roles in ED and KS were investigated through bioinformatics analysis, cellular functional assays are required to elucidate their precise biological functions and molecular mechanisms in disease progression.

## Conclusions

Through integrated analysis of three gene expression datasets related to KS, this study identified a total of 2 biomarkers commonly associated with both KS and AS. In-depth functional analyses indicated that these biomarkers may participate in the pathogenesis of KS by regulating mechanisms such as cell adhesion molecules, focal adhesion, and lysosome pathways. These findings deepen our understanding of the molecular mechanisms underlying KS, particularly uncovering the shared pathological pathways between KS and AS, and provides a theoretical foundation for the development of individualized treatment strategies. Future studies should focus on validating the clinical utility of these biomarkers and exploring the translational potential of the predicted drugs in kidney stone management.

## Supporting information

S1 Table The primer sequences of NEK2, UCHL1, and GAPDH. (DOCX)

S2 Table Differentially expressed genes in the GSE73680 dataset. (XLSX)

S3 Table Differentially expressed genes in the GSE132651 dataset. (XLSX)

S4 Table GSEA enrichment pathways for NEK6. (XLSX)

S5 Table GSEA enrichment pathways for UCHL1. (XLSX)

S6 Table Differentially expressed lncRNAs in the GSE117518 dataset. (XLSX)

## Abbreviations

KS: Kidney stones
ED: Endothelial dysfunction
AS: Atherosclerosis
LASSO: Least absolute shrinkage and selection operator
DEGs: Differentially expressed genes
GSEA: Gene set enrichment analysis
RT-qPCR: Reverse transcription quantitative polymerase chain reaction
AAC: Abdominal aortic calcification
RP: Renal papillary
GSVA: Gene set variation analysis
GO: Gene Ontology
KEGG: Kyoto Encyclopedia of Genes and Genomes
HPA: Human Protein Atlas
UniProt: Universal Protein Resource
GTEx: Genotype-Tissue Expression
ENCORI: ENcyclopedia of RNA InteractiONs
DGIdb: Drug-Gene Interaction Database
TFs: Transcription factors
CTD: Comparative Toxicogenomics Database
ECM: Extracellular matrix
T-reg cells: T-regulatory cells
NK cells: Natural killer cells
