# Supplementary material for: Bioinformatics and experimental studies of atherosclerosis-related endothelial dysfunction genes in renal calculi and exploration of their molecular mechanisms
Source: PLoS One. 2026 Aug 4;21(8):e0354595. doi: 10.1371/journal.pone.0354595 (PMC13436723; doi:10.1371/journal.pone.0354595)
Supplement: Supplementary file 1 [file pone.0354595.s001.docx]

Supplementary Table 1| The primer sequences of NEK2, UCHL1and GAPDH.

| Primer | Sequence(5’-3’) |
| --- | --- |
| NEK2 F | GACGAATGCCTTTTCCGGTG |
| NEK2 R | GACTTCTCCTTGCTCACGCT |
| UCHL1 F | GCTCCCAAAGTGGCTTAGGT |
| UCHL1 R | GCCTCAGGTCTATGAACCCAG |
| GAPDH F | CGAAGGTGGAGTCAACGGATTT |
| GAPDH R | ATGGGTGGAATCATATTGGAAC |
