# Supplementary material for: Bioinformatics and experimental studies of atherosclerosis-related endothelial dysfunction genes in renal calculi and exploration of their molecular mechanisms
Source: PLoS One. 2026 Aug 4;21(8):e0354595. doi: 10.1371/journal.pone.0354595 (PMC13436723; doi:10.1371/journal.pone.0354595)
Supplement: Supplementary file 2 [file pone.0354595.s008.docx]

Response Letter

Dear Editors and Reviews,

We would like to express our sincere gratitude for the time and effort that you have invested in reviewing our manuscript. We found the comments to be highly constructive and helpful in improving the quality and rigor of our work. We have carefully considered every comment and have made corresponding revisions to the manuscript. A point-by-point response to the editor’s and reviewers’ comments is presented below.

**Response to the editor,**

Dear editor,

Thank you very much for the opportunity to revise our manuscript and for your invaluable editorial feedback. The highly insightful comments provided by you have been instrumental in enhancing the clarity and depth of our research. We have addressed all your concerns point-by-point and hope the revised version now meets the high standards of your journal.

Comment 1: Over-interpretation of in-silico outcomes, the identified genes need to be validated by experimental techniques.

Response:

We sincerely appreciate your valuable and professional suggestions. We fully agree with your concern regarding the over-interpretation of our bioinformatics results and the lack of experimental validation for the key candidate genes. Following your advice, we have carefully revised the manuscript to tone down the functional inferences made solely from bioinformatics predictions. We ensured that all conclusions are presented objectively and are strictly supported by the existing data. Additionally, we have added a new paragraph in the Discussion section (Lines 404-413)**.** We explicitly state that NEK2 and UCHL1 are preliminary biomarkers screened via bioinformatics, and our study is limited to expression-level analysis. We acknowledge that further in vitro and in vivo experiments, such as Western blot, immunohistochemistry, and cellular functional assays, are indispensable to elucidate their exact biological functions and molecular mechanisms in disease progression.

Thank you again for your rigorous evaluation, which has greatly strengthened the scientific rigor of our study.

Comment 2: The training cohort (GSE73680) contained 29 KS patient renal papillary (RP) tissue samples and 9 control RP tissue samples, and validation cohort (GSE117518) included 3 KS patient RP tissue samples and 3 control RP tissue samples. The authors should signify the rationale in doing so and selection criteria

Response:

Thank you very much for your valuable comments on the dataset selection and grouping scheme of our study. Regarding your concerns about the rationale for choosing the training and validation cohorts and the sample inclusion criteria, we have supplemented the relevant explanations in the Method and Material section.

The selection of GSE73680 and GSE117518 datasets was primarily based on two criteria. First, there is a high consistency in tissue origins. Both datasets consist of renal papillary (RP) tissues from kidney stone patients and normal controls, which highly matches the specific lesion site focused on in our study, maximizing the homogeneity of the subjects. Second, these datasets have strong literature support and reliability. Both have been applied and validated by previous studies in the field of kidney stones (PMID: 39759764; PMID: 40664942), confirming their data quality and clinical representativeness.

Thanks again for your rigorous review and professional guidance. We have carefully revised and refined the manuscript according to your comments.

Comment 3: Results for UCHL1 need strong statistical significance

Response:

We sincerely appreciate your rigorous comments regarding the statistical significance of the UCHL1 results. We chose to retain UCHL1 as a biomarker based on the following three robust scientific justifications. Firstly, UCHL1 was stably selected by the LASSO screening model, making it a pivotal gene in our model construction. Secondly, the expression trend of UCHL1 is highly consistent between the training set (GSE73680) and the validation set (GSE117518), showing excellent reproducibility. Furthermore, functional annotations indicate that UCHL1 is closely related to cell adhesion, proteasome pathways, and inflammatory responses. Given that these pathways are well-supported by existing literature regarding kidney stone pathogenesis, the inclusion of UCHL1 holds strong biological plausibility.

We completely agree with your statistical observation. Therefore, we have explicitly noted this in the Discussion section (Lines 404-413) of the manuscript. We explain that the failure of UCHL1 to reach statistical significance may be influenced by the small sample size and tissue heterogeneity. Despite this, UCHL1 remains of significant biological importance. We emphasize that expanding the clinical sample size and further validating its expression and function at the protein level are essential next steps. Thank you again for your professional scrutiny and constructive advice.

Comment 4: Reference should be stronger to the manuscript

Response:

Thank you very much for your insightful comments concerning the authority and relevance of the references cited in our study. Following the advice of you and Review 3, we have systematically reviewed, supplemented, and updated the references across the entire manuscript to bolster our theoretical foundation and academic support.

Specifically, we first conducted a rigorous, sentence-by-sentence verification of the Introduction, Results, and Discussion sections to ensure that every core argument is firmly backed by high-quality and highly relevant literature. Secondly, we prioritized citing high-quality research directly pertaining to kidney stones, atherosclerosis, and renal tubular pathology, thereby significantly improving the timeliness and persuasiveness of our theoretical basis.

We are confident that these modifications have greatly enhanced the theoretical depth and scientific rigor of our manuscript. We sincerely appreciate your professional guidance, and we have fully updated the references as required.

**Response to Reviewer 1:**

Dear Reviewer,

Thank you very much for your careful review and invaluable suggestions. Your professional feedback regarding our bioinformatics interpretations, biomarker screening rationale, and statistical analyses have significantly enhanced the scientific rigor and objectivity of our study. We have thoroughly revised the manuscript according to your guidance, particularly by toning down absolute statements and expanding the discussion on study limitations. Please find our detailed, point-by-point responses below.

Comment 1: Overinterpretation of bioinformatic predictions. The manuscript frequently draws conclusions related to molecular mechanisms and functional roles of the identified genes. However, no experimental data are presented to directly support these mechanistic claims. In the absence of functional or mechanistic validation, the findings derived from bioinformatic analyses should be framed strictly as predictions or associations. I strongly recommend extensive rephrasing throughout the manuscript to clearly distinguish predictive bioinformatic insights from experimentally validated molecular mechanisms.

Response:

Thank you very much for your rigorous and professional comments. We fully agree with your criticism regarding the over-interpretation of our bioinformatics predictions and the lack of strict distinction between computational inferences and experimental conclusions.

According to your suggestions, we have toned down absolute statements throughout the text to strictly separate bioinformatics predictions from experimental validations. Specifically, we systematically replaced overly definitive mechanistic terms (e.g., reveal, elucidate, confirm, prove) with more cautious alternatives (e.g., suggest, predict, imply, provide clues for). Similarly, assertive regulatory phrases (e.g., regulate, mediate, participate in) were modified to associative descriptions (e.g., may be associated with, potentially involved in, might act through). Furthermore, the expression differences validated by RT-qPCR are now described purely objectively. For instance, the phrase "GSEA analysis indicates that NEK2 regulates the insulin signaling pathway" has been carefully revised to "GSEA analysis suggests that NEK2 may be associated with the insulin signaling pathway."

By strictly differentiating between bioinformatics predictions and experimental validations throughout the text, the scientific rigor of our manuscript has been substantially improved. We sincerely appreciate your invaluable guidance.

Comment 2: Rationale and scope of experimental validation. The rationale for selecting only UCHL1 and NEK2 for validation is unclear. Given that four candidate biomarkers were identified, it is important to justify why MMP10 and HEY1 were excluded from experimental follow-up. Additionally, it is not clear whether these biomarkers are intended to be relevant only at the transcript level or whether protein-level validation was considered. Validation using immunohistochemistry in patient samples would likely be more clinically relevant and informative, particularly for biomarker studies.

Response:

Thank you sincerely for your insightful comments regarding the rationale and scope of the candidate biomarker validation in our study.

Regarding the rationale for our validation targets, although four candidate biomarkers (NEK2, UCHL1, MMP10, and HEY1) were initially screened via bioinformatics analysis, we ultimately prioritized only NEK2 and UCHL1 for experimental validation. This decision was primarily driven by the fact that NEK2 and UCHL1 maintained highly consistent expression trends and significant differential expression (P < 0.05) across two independent datasets, demonstrating strong reproducibility and stability. Conversely, MMP10 and HEY1 showed inconsistent trends: while differentially expressed in the training set, they failed to reach statistical significance in the validation set. To ensure the reliability of our experimental validation based on the principle of cross-cohort reproducibility, we temporarily excluded MMP10 and HEY1. Their roles may be further evaluated in an independent cohort with larger sample size. Furthermore, we completely agree with your comment regarding the unclear validation dimensions. Accordingly, we have supplemented the manuscript with an explicit limitation statement concerning protein-level validation in the Discussion section (Line 404-413). We clarify that our current work is limited to preliminary validation at the transcriptional level. Future studies are warranted to verify the protein expression of these target genes through techniques such as immunohistochemistry in expanded clinical cohorts, thereby evaluating their true clinical translational value.

We greatly appreciate your professional guidance, which has strengthened our manuscript.

Comment 3: Lack of convincing validation for UCHL1. The qRT-PCR results for UCHL1 do not reach statistical significance. It is therefore difficult to consider this as successful validation of the bioinformatic prediction. The authors should either provide additional supporting data, expand the sample size, or reconsider the strength of the claim regarding UCHL1 as a validated biomarker.

Response:

We sincerely appreciate your rigorous comments regarding the statistical significance of the UCHL1 results. After careful consideration, we chose to retain UCHL1 as a biomarker based on several scientific justifications.

First, UCHL1 was stably selected by the LASSO screening model, making it a pivotal gene in our model construction. Second, its expression trend is completely consistent between the training set and the validation set, indicating excellent reproducibility. Third, functional annotations show that UCHL1 is closely related to cell adhesion, proteasome pathways, and inflammatory responses. These mechanisms that are well-supported by existing literature on kidney stone pathogenesis, thus providing strong biological plausibility.

Additionally, we have transparently acknowledged your valid statistical concern in the Discussion section (Line 404-413). We state that the failure of UCHL1 to reach strict statistical significance may be influenced by the small sample size and tissue heterogeneity. Despite this, UCHL1 remains of significant biological importance. We emphasize that expanding the clinical sample size and further validating its expression and function at the protein level are essential next steps.

Thank you again for your professional scrutiny and constructive advice. We have thoroughly refined the manuscript as requested.

**Response to Reviewer 2:**

Dear Reviewer,

We sincerely thank you for your time, positive evaluation, and constructive feedback. We are highly encouraged by your recommendation that the manuscript can be accepted for publication upon revision. We deeply appreciate your insightful suggestions regarding the inclusion of a global scenario for comparative analysis of the molecular mechanisms, as well as the need for professional English editing and capitalization standardization. We have carefully considered and fully implemented all your valuable advice in the revised manuscript. Our detailed point-by-point responses are provided below.

Comment 1: My suggestion will be to provide a global scenario also with a comparative analysis to understand how the molecular mechanisms differ/not differ (A paragraph highlighting the global scenario would be sufficient).

Response:

We sincerely appreciate your insightful and valuable suggestion. As requested, we have introduced a new paragraph to the manuscript that provides a global context and population comparison. This revision places our research within an international framework, clarifying both the commonalities and the population-specific features of the underlying molecular mechanisms.

Specifically, we highlight that globally, kidney stones and atherosclerosis share high incidence rates and notable population heterogeneity, driven by significant differences in genetic backgrounds, lifestyles, and metabolic profiles across different populations (e.g., Western vs. Asian). Furthermore, we note that international transcriptomic and GWAS studies have consistently identified calcium metabolism disorders, inflammation, oxidative stress, and abnormal cell adhesion as core pathogenic pathways in kidney stones. The high concordance between these global findings and our bioinformatic results strongly reflects the cross-population conservation of these molecular mechanisms. Importantly, we emphasize that while global studies have largely concentrated on classic susceptibility genes (e.g., the SLC family and CLDN14), our study is the first to report NEK2 and UCHL1 as shared molecular biomarkers for both kidney stones and atherosclerosis.

We have incorporated this into the first paragraph of the Discussion section (Line 311-324) in the revised manuscript. We are incredibly grateful for your professional guidance, which has greatly broadened the perspective of our study.

Comment 2: it will be a good idea to take help of a professional for shaping the English language of the manuscript and also take care of the Capital letters in between the sentences. There’s no uniformity. Once done the manuscript can be accepted for publication.

Response:

Thank you very much for your valuable feedback regarding the English language quality and capitalization formatting. The manuscript has undergone comprehensive language polishing by professional editors at Editage to improve fluency and correct any grammatical, structural, or lexical inaccuracies. We have reviewed the entire text to standardize capitalization. Strict rules have been applied so that only sentence initials and proper nouns are capitalized, thoroughly resolving the previous inconsistencies.

We believe these revisions have substantially elevated the manuscript's readability and formatting to meet the journal's standards. Below is the certificate of English editing.

**Response to Reviewer 3:**

Dear Reviewer,

We would like to express our profound gratitude for the exceptional time and effort you invested in reviewing our manuscript. Your highly rigorous, systematic, and insightful comments have been instrumental in elevating the overall quality of our study. We particularly appreciate your meticulous oversight regarding the technical details of our bioinformatics pipeline, the accuracy of our clinical sample data, and the biological interpretation of our findings. Following your expert guidance, we have implemented comprehensive revisions. Each of your points is addressed in detail below, and we hope the revised manuscript now meets your high standards for publication.

Major concern 1: The references in the introduction are either incorrect or inappropriately used to support the text.

We would like to express our deepest gratitude for your rigorous and meticulous review regarding the accuracy and relevance of our references. Regarding the specific issues you identified in the first 10 references, we have verified and corrected each one to ensure that every citation precisely matches the corresponding text. Apart from the 7 references you mentioned, we have taken a step further to conduct a comprehensive and full-coverage verification of all references. By comparing the cited text against the abstracts and core conclusions of the original papers sentence-by-sentence, we guarantee that every citation now provides direct content support and high logical matching, completely eliminating any mismatched citations.

Thank you once again for your scrutiny and invaluable guidance, which have profoundly improved the academic rigor of our study.

Major concern 2: The authors describe the data from GSE73680 as the training cohort and the data from GSE117518 as the validation cohort for this study. The authors should explain what steps, if any, they took to perform batch correction between these datasets. A brief review of these data revealed that a previous study also analyzed these two datasets together and found systematic differences in expression levels due to batch effect (doi: 10.1038/s41598-025-05087-w). The authors should perform a similar analysis and consider citing this paper.

Response:

We sincerely thank you for your professional insight regarding the dataset selection and potential batch effects. We have carefully reviewed the relevant studies you mentioned concerning systemic batch effects between the GSE73680 and GSE117518 datasets. In light of our experimental design, we would like to provide a detailed clarification on why batch effect correction was not required in this specific study.

Our study strictly adheres to an independent validation design. The GSE73680 dataset was used solely for the identification of differentially expressed genes (DEGs), LASSO model construction, and core pathway analysis. The GSE117518 dataset was used only to verify the expression trends and reproducibility of the candidate genes identified from the training set. The fundamental purpose of batch effect correction is to eliminate systemic technical noise when integrating multiple datasets for a combined analysis. Since our workflow processed these datasets independently and did not involve any data merging, the interference of batch effects was inherently bypassed by the experimental design itself. Applying batch correction across non-merged, independent cohorts is unnecessary and could potentially introduce artificial bias into the validation process.

We hope this clarification addresses your concerns regarding the technical rigor of our bioinformatics pipeline. Thank you again for your expert guidance.

Major concern 3: The authors should comment on whether they expect any gene expression differences to exist between the N=23 CaOx kidney stone formers and the N=6 CaP stone formers from GSE73680. These differences in KS presentation should be annotated where appropriate in Fig. 1A-B. Furthermore, how does the present data analysis differ from the analysis that was performed for each original dataset?

Response:

We sincerely appreciate your insightful suggestion regarding the subgroup analysis of stone subtypes within the GSE73680 dataset. Following your advice, we have re-analyzed the samples by grouping them according to stone composition: Calcium Oxalate (CaOx, n = 24), Calcium Phosphate (CaP, n = 5), and Normal Renal Papilla (RP, n = 6) as the control group. The detailed modifications and findings are as follows.

Based on the stone composition metadata in GSE73680, we conducted independent DEA for the CaOx and CaP groups against the normal RP control. We identified 318 up-regulated and 737 down-regulated genes comparing between CaOx group vs. Control group (criteria: |log FC| > 1 and P < 0.05). Using the same criteria, we identified 252 up-regulated and 121 down-regulated genes comparing between CaOx group vs. Control group. To provide a clearer understanding of the molecular characteristics of these two subtypes, we have generated a Venn diagram to visualize the intersection and specificity of the DEGs from both groups. This comparison highlights the shared and unique molecular signatures between CaOx and CaP stone-forming tissues. We have included this Venn diagram as Supplementary Figure with a detailed legend specifying the groups and gene counts. While these subgroup analyses provide valuable insights, the core objective of our study is to explore the general pathological mechanisms of kidney stones rather than a comparative study of CaOx versus CaP subtypes. To maintain the thematic focus and logical flow of the manuscript, we have opted not to incorporate the CaOx/CaP labeling or detailed comparative analysis into Figure 1A-B or the main body of the text. This ensures that the primary research narrative remains concise and targeted.

We believe these additional analyses address your concerns and strengthen the evidence base of our study. Thank you again for your professional guidance.

Major concern 4. The authors should elaborate on how the gene set enrichment analysis was performed. The input to GSEA should be a ranked list of all genes, but Fig. 2D appears to show enrichment of a single gene (either NEK2 or UCHL1). Furthermore, the text describing this figure should distinguish between enrichment and depletion in the pathways, as some have positive enrichment scores while others have negative enrichment scores. Also, the text mentions pathways (i.e., insulin and squamous cell functions) that are not supported by the figure.

We would like to express our sincere gratitude for your rigorous and constructive comments regarding the implementation, visualization, and textual description of the GSEA. We have taken your concerns regarding the GSEA methodology, the distinction between positive and negative enrichment, and the consistency between the text and figures very seriously.

To address your concern that the GSEA input should be a ranked list of all genes rather than individual genes, we have provided a comprehensive description of the GSEA workflow in the Materials and Methods section (Lines 137-145). In response to your observation regarding the lack of distinction between enriched and depleted pathways, we now strictly distinguish between positive and negative enrichment in the Results section and (Lines 248-254). We sincerely apologize for the discrepancies regarding the insulin and squamous cell function pathways mentioned in the previous version. Upon a comprehensive audit, we have removed these two pathways from the text, as they were not shown in the figures. We now exclusively discuss the significant pathways that are explicitly visualized in the figures, ensuring total consistency between the written analysis and the graphical data.

These revisions have clarified the GSEA pipeline, refined the interpretation of enrichment directions, and resolved all textual-graphical inconsistencies. We believe these changes fully address your concerns. Thank you again for your professional guidance.

Major concern 5. The authors should attempt to interpret the findings from the bioinformatics tools that were employed. For example, what relevance, if any, do the following analyses have on identifying biomarkers shared between atherosclerosis and kidney stone formation? i.e., subcellular localization, TF interaction predictions, miRNA/lncRNA predictions, tissue expression, drug prediction. For example, without more information on the mechanism of either biomarker gene in the development or progression of disease, how are the drug interaction predictions useful for the current study?

Response:

We sincerely thank you for your insightful suggestion to further elucidate the biological significance of our bioinformatics findings. We agree that connecting these computational results to the shared biomarkers and pathological links between kidney stones (KS) and atherosclerosis (AS) is crucial. Accordingly, we have substantially revised the Discussion section (Line 365-393) to provide mechanistic interpretations for each analysis. We believe these revisions significantly enhance the interpretative depth and scientific rigor of our study.

Major concern 6. Similarly, the discussion of unrelated disorders that either NEK2 or UCHL1 may be involved in (paragraph 2 of the discussion) does not appear relevant to the current study. I would suggest rewriting this part of the discussion to focus on synthesizing relevant literature on atherosclerosis and kidney stone formation in light of the findings from this study.

Response:

We would like to express our sincere gratitude for your critical and constructive feedback regarding the second paragraph of the Discussion section. We agree that the extensive descriptions of NEK2 and UCHL1 in unrelated diseases detracted from the primary focus of our study. Accordingly, we have removed the mechanistic descriptions of NEK2 and UCHL1 in diseases unrelated to our research scope. Instead, we have added mechanistic descriptions of them in endothelial system or kidney diseases (Discussion section Line 347-364). This adjustment prevents the narrative from drifting away from the central theme. We believe the revised Discussion now more effectively supports our conclusions while maintaining the thematic integrity of the manuscript.

Major concern 7. On line 339 the authors say that "PCR analysis revealed upregulated expression of UCHL1 and NEK2 in stone formers." However, Fig. 6 shows that median UCHL1 expression is actually lower in stone formers and that the difference is not significant. If the expression of UCHL1 could not be validated by RT-qPCR in a third cohort, what utility does this gene have as a biomarker?

Response:

We would like to express our sincere gratitude for your rigorous and constructive critique regarding the RT-qPCR validation results and the positioning of UCHL1 as a biomarker. We have taken your comments concerning the discrepancies between our text and Figure 6, as well as the lack of statistical significance for UCHL1, very seriously. We have performed a word-by-word revision of the manuscript to ensure total transparency and scientific accuracy.

We have re-verified the RT-qPCR data from our clinical samples and thoroughly revised the descriptions in Line 339 and related paragraphs to ensure they strictly align with the data presented in Figure 6. The description has been corrected to. We have maintained the significant validation results for NEK2, ensuring the narrative is supported by the experimental evidence.

To address your concerns regarding the inclusion of UCHL1, we would like clarify its value based on the following scientific rationale. First, despite the lack of transcriptional significance in our specific clinical cohort, UCHL1 was consistently selected by the LASSO regression model and showed a stable trend across two independent public datasets. Second, UCHL1 plays established roles in protein degradation, oxidative stress, and inflammation. These processes are central to both kidney stone formation and endothelial dysfunction. Third, we have explicitly stated the exploratory nature of this finding and noted that larger sample sizes and protein-level validations (e.g., Immunohistochemistry or Western blot) are required in future studies to further elucidate its role.

These revisions have eliminated the inconsistencies between our text and figures while clearly defining the exploratory status of UCHL1. We believe these changes significantly enhance the rigor and transparency of our study.

Minor concern 1. On line 89 in the introduction, the authors say that “Conversely, ED exacerbates renal inflammation…” However, this statement is not the converse of the preceding sentence. I suggest rephrasing, e.g., “ED further exacerbates renal inflammation…”

Response: We have changed the phrase to “ED further exacerbates renal inflammation” according to your suggestion (Line 91 of the revised manuscript). We deeply appreciate your professional guidance on this detail.

Minor concern 2. On line 104 in the data collection section of the methods, the authors say they accessed 29 KS samples and 9 control samples from GSE73680. However, on GEO this study is described as containing only 7 control samples.

Response:

We sincerely thank you for your meticulous review and for pointing out the inaccuracy regarding the sample size description of the GSE73680 dataset. We have carefully re-verified the metadata in the GEO database. We confirm that the GSE73680 dataset indeed comprises 29 kidney stone (KS) samples and **6 control samples**, rather than the 9 control samples previously stated in the manuscript. We deeply apologize for this error in our text.

To rectify this, we have uniformly corrected the data collection description in the Methods section as follows: The training cohort (GSE73680) contained 29 KS patient renal papillary (RP) tissue samples and 6 control RP tissue samples" (Line 106-108). Furthermore, we have conducted a thorough check throughout the entire manuscript to ensure that the reported sample sizes are now strictly consistent with the actual data used in our downstream analyses.

Thank you again for your rigorous oversight, which has helped us ensure the absolute accuracy of our manuscript.

Minor concern 3. Why were different effect sizes thresholds used for identifying differentially expressed genes in GSE73680 (abs. LFC >1) vs. GSE132651 (abs. LFC >0.5)? Does this affect the number of overlapping DEGs found between KS and AS conditions?

Response:

We sincerely thank you for your professional inquiry regarding the criteria for selecting DEGs across different datasets. We have provided a detailed rationale for employing dataset-specific |log FC| thresholds and conducted a sensitivity analysis to demonstrate the robustness of our findings.

The selection of different |log FC| thresholds was strategically based on the inherent biological characteristics and gene expression amplitudes of the respective tissues. The GSE73680 dataset compares stone-forming lesions with normal tissues, where gene expression changes are typically more pronounced. Therefore, a stricter threshold of |log FC| > 1 was applied to ensure high specificity and reliability of the identified DEGs. In contrast, transcriptomic changes in endothelial cells are generally more subtle and exhibit lower fold-change magnitudes in the GSE132651 dataset. To sensitively capture key genes associated with endothelial dysfunction and avoid overlooking biologically significant molecules with smaller effect sizes, we adopted a more inclusive threshold of |log FC| > 0.5. This strategy balances sensitivity and biological relevance across disparate tissue types. To validate our approach, we performed a sensitivity analysis by increasing the threshold for GSE132651 from |log FC| > 0.5 to |log FC| > 1. While the number of DEGs decreased with the stricter threshold, the primary biomarker, NEK2, remained statistically significant (P < 0.05). This demonstrates that while the total number of DEGs is sensitive to threshold adjustments, the core biological significance and the identification of NEK2 are robust and not dependent on a single arbitrary threshold.

In conclusion, maintaining a threshold of |log FC| > 0.5 for the GSE132651 dataset is scientifically justified to preserve key candidate genes and ensure the reliability of our cross-dataset validation. Thank you again for your rigorous review and valuable suggestions.

Minor concern 4. The readability of the methods is poor due to grammatical mistakes

Response:

We sincerely thank you for your valuable feedback regarding the readability and grammatical issues in the Materials and Methods section. We have taken this concern very seriously and have conducted a comprehensive, professional English language polishing of the entire manuscript by professional editors at Editage. Below is certificate of English editing.

Minor concern 5. Is Fig. 1B missing a control sample? Only 6 controls are shown in the heatmap but the GSE73680 dataset has 7 controls.

Response:

We sincerely thank you for your meticulous observation regarding the number of control samples presented in the heatmap **(Figure 1B)**. We have conducted a rigorous verification against the original GEO database records and our internal analytical pipeline.

Upon re-examining the official records of the GSE73680 dataset, we confirmed that the subset utilized for our downstream analysis contains 6 qualified control samples. Our entire study, including differential expression analysis, LASSO modeling, and heatmap generation, was consistently performed using these 6 controls. The heatmap in Figure 1B accurately displays these 6 control samples, which is strictly concordant with the actual data processed in our study. No samples were omitted or excluded from the visualization; the discrepancy previously noted in the text (which has now been corrected to "6" as per Minor concern 2) was a clerical error in the manuscript's description, while the figure itself has always been correct. To further improve the transparency of our study, we have updated the figure legend for Figure 1B to explicitly state the number of samples included and provide brief information regarding the quality control criteria used for sample selection.

We believe this clarification resolves the concerns regarding sample consistency between our analysis and its visualization. Thank you again for your rigorous oversight.

Minor concern 6. The y axis in Fig. 3A is too large. Please select better limits for the y axis. Furthermore, the authors should comment on whether these expression levels are biologically significant. For example, show a comparison to other cell types that are not immune cells.

Response:

We sincerely thank you for your valuable suggestions regarding the graphical representation and biological interpretation of Figure 3A. We have optimized the descriptions and provided additional context to address your concerns.

In response to your suggestion to compare the expression levels in immune cells with other cell types, we have further checked the HPA database. We would like to clarify that the immune cell expression profile in Figure 3A was directly generated from the standardized output of the HPA database. Due to the database's fixed visualization format, the original tool does not support customized modifications to the axis ranges, layout, or the integration of additional non-immune cell types into this specific plot. To address the biological significance without compromising the integrity of the original database output, we have added a detailed explanatory note in the Results and Figure Legends (Lines 266-271). We explicitly state that the expression levels of NEK2 and UCHL1 in immune cells are based on HPA-standardized data. We clarify that NEK2 exhibits moderate-to-high expression levels across multiple immune cell lineages. Furthermore, we have cross-referenced data from other HPA modules (including single-cell type, tissue cell type, and monocyte-derived brain cell datasets) to provide a broader comparative context, confirming that these biomarkers are functionally relevant in both immune and non-immune compartments.

By providing this additional comparative context in the text, we have clarified the biological significance of the expression levels while maintaining the accuracy of the standardized data visualization. Thank you again for your professional guidance.

Minor concern 7. Instead of using lncRNA prediction algorithms, why not use the non-coding RNA microarray data from GSE117518? How do these data compare with the predictions?

Response:

We would like to express our sincere gratitude for your insightful suggestion regarding our lncRNA analysis strategy. Following your advice, we have fully incorporated the non-coding RNA (ncRNA) microarray data from the GSE117518 dataset into our workflow. We have replaced the purely predictive approach with a validated analysis that intersects predicted lncRNAs with actual differentially expressed lncRNAs (DE-lncRNAs).

We re-mined the lncRNA expression matrix from the GSE117518 dataset using the R package "limma" to identify DE-lncRNAs between the kidney stone (KS) group and the normal control group. Applying the thresholds of P < 0.05 and |log FC| > 0.5, we identified a total of 1,625 DE-lncRNAs (1,025 up-regulated and 600 down-regulated, Supplementary Table S6).

To ensure the robustness of our findings, we performed an intersection analysis between the 39 predicted lncRNAs involved in the NEK2 regulatory network and the 1,625 DE-lncRNAs identified from the actual microarray data. This rigorous filtering yielded three consensus candidate lncRNAs: AC108134.2, SNHG15, and MIR4458HG. These three lncRNAs are not only predicted by algorithms to regulate NEK2 but also exhibit significant differential expression in clinical KS samples from the GSE117518 dataset. This dual-level validation significantly enhances the reliability of our lncRNA identification and provides high-confidence core molecules for exploring the NEK2-related ceRNA network in kidney stone pathogenesis. We have incorporated these new findings and the corresponding visualizations into the Results section (Lines 282-288).

We believe that this data-driven refinement, as per your suggestion, has substantially strengthened the lncRNA analysis framework and elevated the overall depth and reliability of our study. Thank you again for your invaluable guidance.

Minor concern 8. Readability of Fig. 5 and Fig. 6 is poor

Response:

We sincerely thank you for your constructive feedback regarding the readability and visual presentation of Figures 5 and 6. We have optimized both figures to meet professional publication standards. We hope that the updated Figures 5 and 6 now provide a much clearer and more effective representation of our findings.

Minor concern 9. The authors should provide the full list of DEGs (p values, effect sizes, etc.) as well as the complete results of the GSEA analysis as supplemental data files.

Response:

We sincerely thank you for your valuable suggestions regarding the data transparency and reproducibility of our study. In accordance with your request, we have organized the complete lists of differentially expressed genes (DEGs) and the full results of the GSEA analysis into supplementary data files.

We have compiled comprehensive tables containing all identified DEGs **(**Supplementary Tables 2-3**).** These tables include essential parameters for each gene, such as Gene Symbols, log2FC, AveExpr, P-values, Adjusted P-values, and the regulation direction. The complete results of our GSEA analysis have been organized into Supplementary Tables 4-5. To ensure maximum verifiability, these tables present all enriched pathways with full statistical details, including Gene Set Names, Enrichment Scores (ES) and Normalized Enrichment Scores (NES), P-values and q-values, and core enrichement gene lists for each enriched pathway.

All aforementioned supplementary files have been submitted alongside the revised manuscript. We believe these additions significantly enhance the transparency and scientific rigor of our research.

Yours sincerely,

Changyan Zhu
