# Supplementary material for: Bioinformatics and experimental studies of atherosclerosis-related endothelial dysfunction genes in renal calculi and exploration of their molecular mechanisms
Source: PLoS One. 2026 Aug 4;21(8):e0354595. doi: 10.1371/journal.pone.0354595 (PMC13436723; doi:10.1371/journal.pone.0354595)
Supplement: Peer review history

# Peer review: Decision Letter 0

**DOI:** 10.1371/journal.pone.0354595.r001

**Authors:** Pramodkumar Gupta

16 Feb 2026

PONE-D-25-68234

Bioinformatics and experimental studies of atherosclerosis-related endothelial dysfunction genes in renal calculi and exploration of their molecular mechanisms

PLOS One

Dear Dr. Zhu,

Thank you for submitting your manuscript to PLOS ONE. After careful consideration, we feel that it has merit but does not fully meet PLOS ONE’s publication criteria as it currently stands. Therefore, we invite you to submit a revised version of the manuscript that addresses the points raised during the review process.

Please submit your revised manuscript by Apr 02 2026 11:59PM. If you will need more time than this to complete your revisions, please reply to this message or contact the journal office at plosone@plos.org. When you're ready to submit your revision, log on to https://www.editorialmanager.com/pone/ and select the 'Submissions Needing Revision' folder to locate your manuscript file.

Please include the following items when submitting your revised manuscript:

- A letter that responds to each point raised by the academic editor and reviewer(s). You should upload this letter as a separate file labeled 'Response to Reviewers'.
- A marked-up copy of your manuscript that highlights changes made to the original version. You should upload this as a separate file labeled 'Revised Manuscript with Track Changes'.
- An unmarked version of your revised paper without tracked changes. You should upload this as a separate file labeled 'Manuscript'.

If you would like to make changes to your financial disclosure, please include your updated statement in your cover letter. Guidelines for resubmitting your figure files are available below the reviewer comments at the end of this letter.

If applicable, we recommend that you deposit your laboratory protocols in protocols.io to enhance the reproducibility of your results. Protocols.io assigns your protocol its own identifier (DOI) so that it can be cited independently in the future. For instructions see: https://journals.plos.org/plosone/s/submission-guidelines#loc-laboratory-protocols. Additionally, PLOS ONE offers an option for publishing peer-reviewed Lab Protocol articles, which describe protocols hosted on protocols.io. Read more information on sharing protocols at https://plos.org/protocols?utm_medium=editorial-email&utm_source=authorletters&utm_campaign=protocols.

We look forward to receiving your revised manuscript.

Kind regards,

Pramodkumar Pyarelal Gupta, PhD

Academic Editor

PLOS One

Journal Requirements:

If the reviewer comments include a recommendation to cite specific previously published works, please review and evaluate these publications to determine whether they are relevant and should be cited. There is no requirement to cite these works unless the editor has indicated otherwise.

Additional Editor Comments (if provided):

Over-interpretation of in-silico outcomes, the identified genes needs to be validated by experimental techniques.

The training cohort (GSE73680) contained 29 KS patient renal papillary (RP) tissue samples and 9 control RP tissue samples, and validation cohort (GSE117518) included 3 KS patient RP tissue samples and 3 control RP tissue samples. The authors should signify the rationale in doing so and selection criteria

Results for UCHL1 need strong statistical significance

Reference should be more strong to the manuscript

[Note: HTML markup is below. Please do not edit.]

Reviewers' comments:

Reviewer's Responses to Questions

**Comments to the Author**

1. Is the manuscript technically sound, and do the data support the conclusions?

The manuscript must describe a technically sound piece of scientific research with data that supports the conclusions. Experiments must have been conducted rigorously, with appropriate controls, replication, and sample sizes. The conclusions must be drawn appropriately based on the data presented.

Reviewer #1: Partly

Reviewer #2: Yes

Reviewer #3: No

**********

2. Has the statistical analysis been performed appropriately and rigorously?

Reviewer #1: Yes

Reviewer #2: Yes

Reviewer #3: No

**********

3. Have the authors made all data underlying the findings in their manuscript fully available?

The PLOS Data policy requires authors to make all data underlying the findings described in their manuscript fully available without restriction, with rare exception (please refer to the Data Availability Statement in the manuscript PDF file). The data should be provided as part of the manuscript or its supporting information, or deposited to a public repository. For example, in addition to summary statistics, the data points behind means, medians and variance measures should be available. If there are restrictions on publicly sharing data—e.g. participant privacy or use of data from a third party—those must be specified.

Reviewer #1: Yes

Reviewer #2: Yes

Reviewer #3: No

**********

4. Is the manuscript presented in an intelligible fashion and written in standard English?

PLOS ONE does not copyedit accepted manuscripts, so the language in submitted articles must be clear, correct, and unambiguous. Any typographical or grammatical errors should be corrected at revision, so please note any specific errors here.

Reviewer #1: Yes

Reviewer #2: No

Reviewer #3: Yes

**********

5. Review Comments to the Author

Please use the space provided to explain your answers to the questions above. You may also include additional comments for the author, including concerns about dual publication, research ethics, or publication ethics. (Please upload your review as an attachment if it exceeds 20,000 characters)

Reviewer #1: In this manuscript, Li et al. describe a bioinformatic analysis aimed at identifying differentially regulated ED-related genes associated with AS in KS. Using in silico approaches, the authors identify four candidate biomarkers (MMP10, UCHL1, NEK2, and HEY1) and further attempt experimental validation of UCHL1 and NEK2 using qRT-PCR. While the bioinformatic workflow is well described and systematically executed, the experimental validation and interpretation substantially weaken the overall conclusions.

Major comments:

1. Overinterpretation of bioinformatic predictions

The manuscript frequently draws conclusions related to molecular mechanisms and functional roles of the identified genes. However, no experimental data are presented to directly support these mechanistic claims. In the absence of functional or mechanistic validation, the findings derived from bioinformatic analyses should be framed strictly as predictions or associations. I strongly recommend extensive rephrasing throughout the manuscript to clearly distinguish predictive bioinformatic insights from experimentally validated molecular mechanisms.

2. Rationale and scope of experimental validation

The rationale for selecting only UCHL1 and NEK2 for validation is unclear. Given that four candidate biomarkers were identified, it is important to justify why MMP10 and HEY1 were excluded from experimental follow-up. Additionally, it is not clear whether these biomarkers are intended to be relevant only at the transcript level or whether protein-level validation was considered. Validation using immunohistochemistry (IHC) in patient samples would likely be more clinically relevant and informative, particularly for biomarker studies.

3. Lack of convincing validation for UCHL1

The qRT-PCR results for UCHL1 do not reach statistical significance. It is therefore difficult to consider this as successful validation of the bioinformatic prediction. The authors should either provide additional supporting data, expand the sample size, or reconsider the strength of the claim regarding UCHL1 as a validated biomarker.

Reviewer #2: The manuscript explains how authors have identified two potential biomarkers that are associated with Kidney Stones and Atherosclerosis in Chinese people.

My suggestion will be to provide a global scenario also with a comparative analysis to understand how the molecular mechanisms differ/not differ (A paragraph highlighting the global scenario would be sufficient).

Also it will be a good idea to take help of a professional for shaping the English language of the manuscript and also take care of the Capital letters in between the sentences. There’s no uniformity. Once done the manuscript can be accepted for publication.

Reviewer #3: Summary

The authors present a joint analysis of three publicly available gene expression microarray studies of either kidney stone formers or patients with coronary endothelial dysfunction (atherosclerosis). The authors identify differentially expressed genes from these datasets, comparing the affected tissues to control samples. They find that 22 DEGs intersect between the kidney stone and atherosclerosis datasets, and, using a LASSO regression model and a validation dataset, narrow their focus to two candidate biomarker genes. The authors then use a series of bioinformatics tools to characterize these two genes and infer a possible connection between kidney stone formation and atherosclerosis. However, the manuscript lacks a convincing interpretation of these findings and has technical problems with the analysis. Furthermore, there appear to be issues with the references (detailed below) that call into question the rigor of the subsequent analysis and conclusions. In the current state, the manuscript is not fit for publication.

Major

1. The references in the introduction are either incorrect or inappropriately used to support the text. Although I found the introduction to be well written and believe it could be factually correct, the misuse of references forces me to question the results and conclusions of the study. The authors must examine the references carefully and fix any mistakes. I reviewed the first 10 references (in the introduction) and found the following issues. Presumably there are more incorrect references, but I stopped after the first 10.

- Reference 2 has the wrong DOI listed – currently it links to a letter to the editor in reference to the intended reference (doi: 10.1016/j.eururo.2012.03.052)

- Reference 3 is used to justify the statistic that kidney stone prevalence is 7.54% in China; this statistic is actually from a different reference (doi: 10.1038/srep41630)

- Reference 4 is used to support a sentence describing the pathogenic mechanisms underlying KS; however, this reference does not discuss these mechanisms

- Reference 5 is used to support a discussion of the challenges in managing KS; however, reference 5 does not discuss kidney stones

- Reference 7 is used to support a discussion of the potential links between KS and AS; however reference 7 does not discuss AS

- Reference 8 is used to support a finding that KS patients have twofold higher aortic calcification; this statistic actually from a different reference (doi: 10.1007/s11255-021-03058-4)

- Reference 9 does not discuss kidney stones

2. The authors describe the data from GSE73680 as the training cohort and the data from GSE117518 as the validation cohort for this study. The authors should explain what steps, if any, they took to perform batch correction between these datasets. A brief review of these data revealed that a previous study also analyzed these two datasets together and found systematic differences in expression levels due to batch effect (doi: 10.1038/s41598-025-05087-w). The authors should perform a similar analysis and consider citing this paper.

3. The authors should comment on whether they expect any gene expression differences to exist between the N=23 CaOx kidney stone formers and the N=6 CaP stone formers from GSE73680. These differences in KS presentation should be annotated where appropriate in Fig. 1A-B. Furthermore, how does the present data analysis differ from the analysis that was performed for each original dataset?

4. The authors should elaborate on how the gene set enrichment analysis was performed. The input to GSEA should be a ranked list of all genes, but Fig. 2D appears to show enrichment of a single gene (either NEK2 or UCHL1). Furthermore, the text describing this figure should distinguish between enrichment and depletion in the pathways, as some have positive enrichment scores while others have negative enrichment scores. Also, the text mentions pathways (i.e., insulin and squamous cell functions) that are not supported by the figure.

5. The authors should attempt to interpret the findings from the bioinformatics tools that were employed. For example, what relevance, if any, do the following analyses have on identifying biomarkers shared between atherosclerosis and kidney stone formation? i.e., subcellular localization, TF interaction predictions, miRNA/lncRNA predictions, tissue expression, drug prediction. For example, without more information on the mechanism of either biomarker gene in the development or progression of disease, how are the drug interaction predictions useful for the current study?

6. Similarly, the discussion of unrelated disorders that either NEK2 or UCHL1 may be involved in (paragraph 2 of the discussion) does not appear relevant to the current study. I would suggest rewriting this part of the discussion to focus on synthesizing relevant literature on atherosclerosis and kidney stone formation in light of the findings from this study.

7. On line 339 the authors say that "PCR analysis revealed upregulated expression of UCHL1 and NEK2 in stone formers." However, Fig. 6 shows that median UCHL1 expression is actually lower in stone formers and that the difference is not significant. If the expression of UCHL1 could not be validated by RT-qPCR in a third cohort, what utility does this gene have as a biomarker?

Minor

1. On line 89 in the introduction, the authors say that “Conversely, ED exacerbates renal inflammation…” However, this statement is not the converse of the preceding sentence. I suggest rephrasing, e.g., “ED further exacerbates renal inflammation…”

2. On line 104 in the data collection section of the methods, the authors say they accessed 29 KS samples and 9 control samples from GSE73680. However, on GEO this study is described as containing only 7 control samples.

3. Why were different effect sizes thresholds used for identifying differentially expressed genes in GSE73680 (abs. LFC >1) vs. GSE132651 (abs. LFC >0.5)? Does this affect the number of overlapping DEGs found between KS and AS conditions?

4. The readability of the methods is poor due to grammatical mistakes

5. Is Fig. 1B missing a control sample? Only 6 controls are shown in the heatmap but the GSE73680 dataset has 7 controls.

6. The y axis in Fig. 3A is too large. Please select better limits for the y axis. Furthermore, the authors should comment on whether these expression levels are biologically significant. For example, show a comparison to other cell types that are not immune cells.

7. Instead of using lncRNA prediction algorithms, why not use the non-coding RNA microarray data from GSE117518? How do these data compare with the predictions?

8. Readability of Fig. 5 and Fig. 6 is poor

9. The authors should provide the full list of DEGs (p values, effect sizes, etc.) as well as the complete results of the GSEA analysis as supplemental data files

**********

6. PLOS authors have the option to publish the peer review history of their article (what does this mean?). If published, this will include your full peer review and any attached files.

If you choose “no”, your identity will remain anonymous but your review may still be made public.

**Do you want your identity to be public for this peer review?**  For information about this choice, including consent withdrawal, please see our Privacy Policy.

Reviewer #1: No

Reviewer #2: **Yes:** Dr. Gauri Awasthi

Reviewer #3: No

**********

[NOTE: If reviewer comments were submitted as an attachment file, they will be attached to this email and accessible via the submission site. Please log into your account, locate the manuscript record, and check for the action link "View Attachments". If this link does not appear, there are no attachment files.]

To ensure your figures meet our technical requirements, please review our figure guidelines: https://journals.plos.org/plosone/s/figures

You may also use PLOS’s free figure tool, NAAS, to help you prepare publication quality figures: https://journals.plos.org/plosone/s/figures#loc-tools-for-figure-preparation.

NAAS will assess whether your figures meet our technical requirements by comparing each figure against our figure specifications.

# Author comment: Author response to Decision Letter 1

**DOI:** 10.1371/journal.pone.0354595.r002

1 Apr 2026

Dear Editors and Reviews,

We would like to express our sincere gratitude for the time and effort that you have invested in reviewing our manuscript. We found the comments to be highly constructive and helpful in improving the quality and rigor of our work. We have carefully considered every comment and have made corresponding revisions to the manuscript. A point-by-point response to the editor’s and reviewers’ comments is presented below.

Response to the editor,

Dear editor,

Thank you very much for the opportunity to revise our manuscript and for your invaluable editorial feedback. The highly insightful comments provided by you have been instrumental in enhancing the clarity and depth of our research. We have addressed all your concerns point-by-point and hope the revised version now meets the high standards of your journal.

Comment 1: Over-interpretation of in-silico outcomes, the identified genes need to be validated by experimental techniques.

Response:

We sincerely appreciate your valuable and professional suggestions. We fully agree with your concern regarding the over-interpretation of our bioinformatics results and the lack of experimental validation for the key candidate genes. Following your advice, we have carefully revised the manuscript to tone down the functional inferences made solely from bioinformatics predictions. We ensured that all conclusions are presented objectively and are strictly supported by the existing data. Additionally, we have added a new paragraph in the Discussion section (Lines 404-413). We explicitly state that NEK2 and UCHL1 are preliminary biomarkers screened via bioinformatics, and our study is limited to expression-level analysis. We acknowledge that further in vitro and in vivo experiments, such as Western blot, immunohistochemistry, and cellular functional assays, are indispensable to elucidate their exact biological functions and molecular mechanisms in disease progression.

Thank you again for your rigorous evaluation, which has greatly strengthened the scientific rigor of our study.

Comment 2: The training cohort (GSE73680) contained 29 KS patient renal papillary (RP) tissue samples and 9 control RP tissue samples, and validation cohort (GSE117518) included 3 KS patient RP tissue samples and 3 control RP tissue samples. The authors should signify the rationale in doing so and selection criteria

Response:

Thank you very much for your valuable comments on the dataset selection and grouping scheme of our study. Regarding your concerns about the rationale for choosing the training and validation cohorts and the sample inclusion criteria, we have supplemented the relevant explanations in the Method and Material section.

The selection of GSE73680 and GSE117518 datasets was primarily based on two criteria. First, there is a high consistency in tissue origins. Both datasets consist of renal papillary (RP) tissues from kidney stone patients and normal controls, which highly matches the specific lesion site focused on in our study, maximizing the homogeneity of the subjects. Second, these datasets have strong literature support and reliability. Both have been applied and validated by previous studies in the field of kidney stones (PMID: 39759764; PMID: 40664942), confirming their data quality and clinical representativeness.

Thanks again for your rigorous review and professional guidance. We have carefully revised and refined the manuscript according to your comments.

Comment 3: Results for UCHL1 need strong statistical significance

Response:

We sincerely appreciate your rigorous comments regarding the statistical significance of the UCHL1 results. We chose to retain UCHL1 as a biomarker based on the following three robust scientific justifications. Firstly, UCHL1 was stably selected by the LASSO screening model, making it a pivotal gene in our model construction. Secondly, the expression trend of UCHL1 is highly consistent between the training set (GSE73680) and the validation set (GSE117518), showing excellent reproducibility. Furthermore, functional annotations indicate that UCHL1 is closely related to cell adhesion, proteasome pathways, and inflammatory responses. Given that these pathways are well-supported by existing literature regarding kidney stone pathogenesis, the inclusion of UCHL1 holds strong biological plausibility.

We completely agree with your statistical observation. Therefore, we have explicitly noted this in the Discussion section (Lines 404-413) of the manuscript. We explain that the failure of UCHL1 to reach statistical significance may be influenced by the small sample size and tissue heterogeneity. Despite this, UCHL1 remains of significant biological importance. We emphasize that expanding the clinical sample size and further validating its expression and function at the protein level are essential next steps. Thank you again for your professional scrutiny and constructive advice.

Comment 4: Reference should be stronger to the manuscript

Response:

Thank you very much for your insightful comments concerning the authority and relevance of the references cited in our study. Following the advice of you and Review 3, we have systematically reviewed, supplemented, and updated the references across the entire manuscript to bolster our theoretical foundation and academic support.

Specifically, we first conducted a rigorous, sentence-by-sentence verification of the Introduction, Results, and Discussion sections to ensure that every core argument is firmly backed by high-quality and highly relevant literature. Secondly, we prioritized citing high-quality research directly pertaining to kidney stones, atherosclerosis, and renal tubular pathology, thereby significantly improving the timeliness and persuasiveness of our theoretical basis.

We are confident that these modifications have greatly enhanced the theoretical depth and scientific rigor of our manuscript. We sincerely appreciate your professional guidance, and we have fully updated the references as required.

Response to Reviewer 1:

Dear Reviewer,

Thank you very much for your careful review and invaluable suggestions. Your professional feedback regarding our bioinformatics interpretations, biomarker screening rationale, and statistical analyses have significantly enhanced the scientific rigor and objectivity of our study. We have thoroughly revised the manuscript according to your guidance, particularly by toning down absolute statements and expanding the discussion on study limitations. Please find our detailed, point-by-point responses below.

Comment 1: Overinterpretation of bioinformatic predictions. The manuscript frequently draws conclusions related to molecular mechanisms and functional roles of the identified genes. However, no experimental data are presented to directly support these mechanistic claims. In the absence of functional or mechanistic validation, the findings derived from bioinformatic analyses should be framed strictly as predictions or associations. I strongly recommend extensive rephrasing throughout the manuscript to clearly distinguish predictive bioinformatic insights from experimentally validated molecular mechanisms.

Response:

Thank you very much for your rigorous and professional comments. We fully agree with your criticism regarding the over-interpretation of our bioinformatics predictions and the lack of strict distinction between computational inferences and experimental conclusions.

According to your suggestions, we have toned down absolute statements throughout the text to strictly separate bioinformatics predictions from experimental validations. Specifically, we systematically replaced overly definitive mechanistic terms (e.g., reveal, elucidate, confirm, prove) with more cautious alternatives (e.g., suggest, predict, imply, provide clues for). Similarly, assertive regulatory phrases (e.g., regulate, mediate, participate in) were modified to associative descriptions (e.g., may be associated with, potentially involved in, might act through). Furthermore, the expression differences validated by RT-qPCR are now described purely objectively. For instance, the phrase "GSEA analysis indicates that NEK2 regulates the insulin signaling pathway" has been carefully revised to "GSEA analysis suggests that NEK2 may be associated with the insulin signaling pathway."

By strictly differentiating between bioinformatics predictions and experimental validations throughout the text, the scientific rigor of our manuscript has been substantially improved. We sincerely appreciate your invaluable guidance.

Comment 2: Rationale and scope of experimental validation. The rationale for selecting only UCHL1 and NEK2 for validation is unclear. Given that four candidate biomarkers were identified, it is important to justify why MMP10 and HEY1 were excluded from experimental follow-up. Additionally, it is not clear whether these biomarkers are intended to be relevant only at the transcript level or whether protein-level validation was considered. Validation using immunohistochemistry in patient samples would likely be more clinically relevant and informative, particularly for biomarker studies.

Response:

Thank you sincerely for your insightful comments regarding the rationale and scope of the candidate biomarker validation in our study.

Regarding the rationale for our validation targets, although four candidate biomarkers (NEK2, UCHL1, MMP10, and HEY1) were initially screened via bioinformatics analysis, we ultimately prioritized only NEK2 and UCHL1 for experimental validation. This decision was primarily driven by the fact that NEK2 and UCHL1 maintained highly consistent expression trends and significant differential expression (P < 0.05) across two independent datasets, demonstrating strong reproducibility and stability. Conversely, MMP10 and HEY1 showed inconsistent trends: while differentially expressed in the training set, they failed to reach statistical significance in the validation set. To ensure the reliability of our experimental validation based on the principle of cross-cohort reproducibility, we temporarily excluded MMP10 and HEY1. Their roles may be further evaluated in an independent cohort with larger sample size. Furthermore, we completely agree with your comment regarding the unclear validation dimensions. Accordingly, we have supplemented the manuscript with an explicit limitation statement concerning protein-level validation in the Discussion section (Line 404-413). We clarify that our current work is limited to preliminary validation at the transcriptional level. Future studies are warranted to verify the protein expression of these target genes through techniques such as immunohistochemistry in expanded clinical cohorts, thereby evaluating their true clinical translational value.

We greatly appreciate your professional guidance, which has strengthened our manuscript.

Comment 3: Lack of convincing validation for UCHL1. The qRT-PCR results for UCHL1 do not reach statistical significance. It is therefore difficult to consider this as successful validation of the bioinformatic prediction. The authors should either provide additional supporting data, expand the sample size, or reconsider the strength of the claim regarding UCHL1 as a validated biomarker.

Response:

We sincerely appreciate your rigorous comments regarding the statistical significance of the UCHL1 results. After careful consideration, we chose to retain UCHL1 as a biomarker based on several scientific justifications.

First, UCHL1 was stably selected by the LASSO screening model, making it a pivotal gene in our model construction. Second, its expression trend is completely consistent between the training set and the validation set, indicating excellent reproducibility. Third, functional annotations show that UCHL1 is closely related to cell adhesion, proteasome pathways, and inflammatory responses. These mechanisms that are well-supported by existing literature on kidney stone pathogenesis, thus providing strong biological plausibility.

Additionally, we have transparently acknowledged your valid statistical concern in the Discussion section (Line 404-413). We state that the failure of UCHL1 to reach strict statistical significance may be influenced by the small sample size and tissue heterogeneity. Despite this, UCHL1 remains of significant biological importance. We emphasize that expanding the clinical sample size and further validating its expression and function at the protein level are essential next steps.

Thank you again for your professional scrutiny and constructive advice. We have thoroughly refined the manuscript as requested.

Response to Reviewer 2:

Dear Reviewer,

We sincerely thank you for your time, positive evaluation, and constructive feedback. We are highly encouraged by your recommendation that the manuscript can be accepted for publication upon revision. We deeply appreciate your insightful suggestions regarding the inclusion of a global scenario for comparative analysis of the molecular mechanisms, as well as the need for professional English editing and capitalization standardization. We have carefully considered and fully implemented all your valuable advice in the revised manuscript. Our detailed point-by-point responses are provided below.

Comment 1: My suggestion will be to provide a global scenario also with a comparative analysis to understand how the molecular mechanisms differ/not differ (A paragraph highlighting the global scenario would be sufficient).

Response:

We sincerely appreciate your insightful and valuable suggestion. As requested, we have introduced a new paragraph to the manuscript that provides a global context and population comparison. This revision places our research within an international framework, clarifying both the commonalities and the population-specific features of the underlying molecular mechanisms.

Specifically, we highlight that globally, kidney stones and atherosclerosis share high incidence rates and notable population heterogeneity, driven by significant differences in genetic backgrounds, lifestyles, and metabolic profiles across different populations (e.g., Western vs. Asian). Furthermore, we note that international transcriptomic and GWAS studies have consistently identified calcium metabolism disorders, inflammation, oxidative stress, and abnormal cell adhesion as core pathogenic pathways in kidney stones. The high concordance between these global findings and our bioinformatic results strongly reflects the cross-population conservation of these molecular mechanisms. Importantly, we emphasize that while global studies have largely concentrated on classic susceptibility genes (e.g., the SLC family and CLDN14), our study is the first to report NEK2 and UCHL1 as shared molecular biomarkers for both kidney stones and atherosclerosis.

We have incorporated this into the first paragraph of the Discussion section (Line 311-324) in the revised manuscript. We are incredibly grateful for your professional guidance, which has greatly broadened the perspective of our study.

Comment 2: it will be a good idea to take help of a professional for shaping the English language of the manuscript and also take care of the Capital letters in between the sentences. There’s no uniformity. Once done the manuscript can be accepted for publication.

Response:

Thank you very much for your valuable feedback regarding the English language quality and capitalization formatting. The manuscript has undergone comprehensive language polishing by professional editors at Editage to improve fluency and correct any grammatical, structural, or lexical inaccuracies. We have reviewed the entire text to standardize capitalization. Strict rules have been applied so that only sentence initials and proper nouns are capitalized, thoroughly resolving the previous inconsistencies.

We believe these revisions have substantially elevated the manuscript's readability and formatting to meet the journal's standards. Below is the certificate of English editing.

Response to Reviewer 3:

Dear Reviewer,

We would like to express our profound gratitude for the exceptional time and effort you invested in reviewing our manuscript. Your highly rigorous, systematic, and insightful comments have been instrumental in e

Attachment Submitted filename: Response letter to the editor and reviewers.docx

# Peer review: Decision Letter 1

**DOI:** 10.1371/journal.pone.0354595.r003

**Authors:** Patrick Goymer

9 Jul 2026

<p>Bioinformatics and experimental studies of atherosclerosis-related endothelial dysfunction genes in renal calculi and exploration of their molecular mechanisms

PONE-D-25-68234R1

Dear Dr. Zhu,

We’re pleased to inform you that your manuscript has been judged scientifically suitable for publication and will be formally accepted for publication once it meets all outstanding technical requirements.

Within one week, you’ll receive an e-mail detailing the required amendments. When these have been addressed, you’ll receive a formal acceptance letter and your manuscript will be scheduled for publication.

An invoice will be generated when your article is formally accepted. Please note, if your institution has a publishing partnership with PLOS and your article meets the relevant criteria, all or part of your publication costs will be covered. Please make sure your user information is up-to-date by logging into Editorial Manager at Editorial Manager® and clicking the ‘Update My Information' link at the top of the page. For questions related to billing, please contact billing support.

If your institution or institutions have a press office, please notify them about your upcoming paper to help maximize its impact. If they’ll be preparing press materials, please inform our press team as soon as possible -- no later than 48 hours after receiving the formal acceptance. Your manuscript will remain under strict press embargo until 2 pm Eastern Time on the date of publication. For more information, please contact onepress@plos.org.

Kind regards,

Patrick Goymer

Staff Editor

PLOS One

Additional Editor Comments (optional):

Reviewers' comments:

Reviewer's Responses to Questions

**Comments to the Author**

1. If the authors have adequately addressed your comments raised in a previous round of review and you feel that this manuscript is now acceptable for publication, you may indicate that here to bypass the “Comments to the Author” section, enter your conflict of interest statement in the “Confidential to Editor” section, and submit your "Accept" recommendation.

Reviewer #2: All comments have been addressed

Reviewer #3: All comments have been addressed

**********

2. Is the manuscript technically sound, and do the data support the conclusions?

The manuscript must describe a technically sound piece of scientific research with data that supports the conclusions. Experiments must have been conducted rigorously, with appropriate controls, replication, and sample sizes. The conclusions must be drawn appropriately based on the data presented.

Reviewer #2: Yes

Reviewer #3: Yes

**********

3. Has the statistical analysis been performed appropriately and rigorously?

Reviewer #2: Yes

Reviewer #3: Yes

**********

4. Have the authors made all data underlying the findings in their manuscript fully available?

The PLOS Data policy requires authors to make all data underlying the findings described in their manuscript fully available without restriction, with rare exception (please refer to the Data Availability Statement in the manuscript PDF file). The data should be provided as part of the manuscript or its supporting information, or deposited to a public repository. For example, in addition to summary statistics, the data points behind means, medians and variance measures should be available. If there are restrictions on publicly sharing data—e.g. participant privacy or use of data from a third party—those must be specified.

Reviewer #2: Yes

Reviewer #3: Yes

**********

5. Is the manuscript presented in an intelligible fashion and written in standard English?

PLOS ONE does not copyedit accepted manuscripts, so the language in submitted articles must be clear, correct, and unambiguous. Any typographical or grammatical errors should be corrected at revision, so please note any specific errors here.

Reviewer #2: Yes

Reviewer #3: Yes

**********

6. Review Comments to the Author

Please use the space provided to explain your answers to the questions above. You may also include additional comments for the author, including concerns about dual publication, research ethics, or publication ethics. (Please upload your review as an attachment if it exceeds 20,000 characters)

Reviewer #2: The manuscript is ready for publication as the comments are answered by the authors with clarity. Recommended for publication now.

Reviewer #3: The authors have provided satisfactory responses to my comments. I thank the reviewers for their efforts in revising the manuscript and agree with their assessment that the revised manuscript is significantly stronger now. I appreciate their thoughtful and comprehensive consideration of my comments.

One final request to the authors is to please indicate whether AI tools were used for this study or in drafting the manuscript.

**********

7. PLOS authors have the option to publish the peer review history of their article (what does this mean?). If published, this will include your full peer review and any attached files.

If you choose “no”, your identity will remain anonymous but your review may still be made public.

**Do you want your identity to be public for this peer review?**  For information about this choice, including consent withdrawal, please see our Privacy Policy.

Reviewer #2: **Yes:** Dr. Gauri Awasthi

Reviewer #3: No

**********

# Editor report: Acceptance letter

**DOI:** 10.1371/journal.pone.0354595.r004

**Authors:** Patrick Goymer

PONE-D-25-68234R1

PLOS One

Dear Dr. Zhu,

I'm pleased to inform you that your manuscript has been deemed suitable for publication in PLOS One. Congratulations! Your manuscript is now being handed over to our production team.

At this stage, our production department will prepare your paper for publication. This includes ensuring the following:

* All references, tables, and figures are properly cited

* All relevant supporting information is included in the manuscript submission,

* There are no issues that prevent the paper from being properly typeset

You will receive further instructions from the production team, including instructions on how to review your proof when it is ready. Please keep in mind that we are working through a large volume of accepted articles, so please give us a few days to review your paper and let you know the next and final steps.

Lastly, if your institution or institutions have a press office, please let them know about your upcoming paper now to help maximize its impact. If they'll be preparing press materials, please inform our press team within the next 48 hours. Your manuscript will remain under strict press embargo until 2 pm Eastern Time on the date of publication. For more information, please contact onepress@plos.org.

You will receive an invoice from PLOS for your publication fee after your manuscript has reached the completed accept phase. If you receive an email requesting payment before acceptance or for any other service, this may be a phishing scheme. Learn how to identify phishing emails and protect your accounts at https://explore.plos.org/phishing.

If we can help with anything else, please email us at customercare@plos.org.

Thank you for submitting your work to PLOS ONE and supporting open access.

Kind regards,

PLOS ONE Editorial Office Staff

on behalf of

Dr Patrick Goymer

Staff Editor

PLOS One
